# The Gene Coexpression Analysis Identifies Functional Modules Dynamically Changed After Traumatic Brain Injury

**DOI:** 10.1155/2021/5511598

**Published:** 2021-04-16

**Authors:** Zhi-jie Zhao, Dong-po Wei, Rui-zhe Zheng, Tinghua Peng, Xiang Xiao, Fu-sheng Li

**Affiliations:** ^1^Department of Neurosurgery, Tongren Hospital, Shanghai Jiao Tong University School of Medicine, Shanghai, China; ^2^Department of Neurosurgery, Shanghai Ninth People's Hospital, Shanghai Jiao Tong University School of Medicine, Shanghai, China; ^3^Department of Critical Care Medicine, Shanghai General Hospital, Shanghai Jiao Tong University School of Medicine, Shanghai, China; ^4^Department of Neurosurgery, Jiangxi Provincial People's Hospital Affiliated to Nanchang University, Nanchang, Jiangxi, China

## Abstract

Traumatic brain injury (TBI) is a major cause of morbidity and mortality, both in adult and pediatric populations. However, the dynamic changes of gene expression profiles following TBI have not been fully understood. In this study, we identified the differentially expressed genes (DEGs) following TBI. Remarkably, Serpina3n, Asf1b, Folr1, LOC100366216, Clec12a, Olr1, Timp1, Hspb1, Lcn2, and Spp1 were identified as the top 10 with the highest statistical significance. The weighted gene coexpression analysis (WGCNA) identified 12 functional modules from the DEGs, which showed specific expression patterns over time and were characterized by enrichment analysis. Specifically, the black and turquoise modules were mainly involved in energy metabolism and protein translation. The green yellow and yellow modules including Hmox1, Mif, Anxa2, Timp1, Gfap, Cd9, Gja1, Pdpn, and Gpx1 were related to response to wounding, indicating that expression of these genes such as Hmox1, Anxa2, and Timp1 could protect the brains from brain injury. The green yellow module highlighted genes involved in microglial cell activation such as Tyrobp, Cx3cr1, Grn, Trem2, C1qa, and Aif1, suggesting that these genes were responsible for the inflammatory response caused by TBI. The upregulation of these genes has been validated in an independent dataset. These results indicated that the key genes in microglia cell activation may serve as a promising therapeutic target for TBI. In summary, the present study provided a full view of the dynamic gene expression changes following TBI.

## 1. Introduction

Traumatic brain injury (TBI) is often caused by a sudden trauma to the head, but its deleterious effects on patients can be life-long and dynamic [1, 2]. It is a highlighted cause of mortality and long-term disability worldwide, and a well-recognized risk factor for neurological and psychological disorders, such as Alzheimer's disease, chronic traumatic encephalopathy, depression, and psychosis [3, 4]. Such chronic consequences induced by TBI are now posing a huge burden for both TBI survivors and the society, yet establishing therapeutic intervention for the progression of tissue damage and neurodegeneration in chronic TBI remains a challenge, as our knowledge of long-term and ever-evolving pathogenic mechanisms behind post-TBI consequences is very limited.

It is believed that chronic traumatic brain inflammation plays a critical role in post-TBI neurodegeneration, and efforts are made to address metabolomic, proteomic, and genomic changes across a series of post-TBI intervals, in hopes of identifying potential long-term biomarkers for defining the injury progression [2, 5, 6]. A previous study has provided temporal profiles of 12 biomarkers in body fluids of TBI patients, demonstrated an association between the severity of TBI and the peak heights of each molecules, and suggested that release mechanisms may vary among different types of molecules, which further hints that successive measurements are essential for TBI diagnosis [7]. Utilizing microarray analysis, a recent study has described abnormal expression of immune mediators and brain injury-induced factors, along with regenerative immunoregulatory genes, in animal models of TBI, and remarked that such dysregulation of gene expression can be long-lasting after the initial injuries [8]. Also in experimental TBI, high levels of expression of Serpina3n, an astroglial activation marker, are found in neurons in the early stage of the injury [9]. Meanwhile, it is observed in another study that the knockout of Serpina3n led to increased neuronal apoptosis in neurons, and that an MMP2-specific inhibitor might serve as a therapeutic target for neurotrauma [10]. In addition, Trem2 and Tyrobp have been identified as major regulators and therapeutic targets in TBI [11, 12]. In the present study, to better capture the dynamics of gene expression and related biological functions at successive post-TBI time points, we conducted differential expression analysis and coexpression analysis to identify the coexpression modules, and characterized their functionalities by overrepresentation enrichment analysis, anticipating to reveal some critical genes involved in the dynamic changes post-TBI.

## 2. Materials and Methods

### 2.1. Gene Expression Data

The gene expression data of cortex tissues were downloaded from Gene Expression Omnibus (GEO) with accession GSE111452 [8]. A total of 60 samples were collected for the analyses in this study. The expression values were normalized to the 75th percentile intensity. Moreover, the gene expression data for validation was obtained from GEO with accession GSE79441 [13] and normalized to fragment per kilo-million (FPKM).

### 2.2. Differentially Expressed Genes

The differential expression analysis was conducted by pairwise comparison for the control, sham, and TBI samples at each time point. The FPKM-based gene expression data was logarithm-transformed. For each comparison, The R limma package was employed to identify the differentially expressed genes [14]. The *P* values were adjusted to the false discovery rate (FDR) to avoid false positives by multiple statistical testing.

### 2.3. Coexpression Network and Functional Modules

The weighted gene coexpression network analysis (WGCNA) was used to identify the functional modules [15]. Specifically, the lowest soft-thresholding power for which the scale-free topology fit was selected. The topological overlap matrix (TOM) similarity was calculated using the power and expression data of differentially expressed genes.

### 2.4. Overrepresentation Enrichment Analysis (ORA)

The overrepresentation enrichment analysis was employed to identify the biological process of Gene Ontology (GO) enriched by the genes within the functional module. The hypergeometric test was used to calculate the *P* value for each GO term, and was implemented in the R clusterProfiler package [16].

### 2.5. Random Forest Classifier

The expression levels of the hub genes for the 7 functional modules were used for the classifier training and validation. The training and validation sets were randomly divided and were used to build the classifier and evaluate the predictive performance of the classifier. The model construction and prediction were implemented by the R e1071 package (https://cran.r-project.org/web/packages/e1071/index.html).

### 2.6. Statistical Analyses

The two-sample and multisample comparisons were tested by the Student *t*-test and analysis of variance (ANOVA), respectively. The Spearman correlations and corresponding *P* values between the trait and hub genes were calculated to evaluate the module-trait relationship. The Kolmogorov-Smirnov test was used to test the enrichment of the six microglial cell activation-related genes in the upregulated genes by TBI.

## 3. Results

### 3.1. Identification of Genes Dysregulated by Traumatic Brain Injury

**Figure 1:**
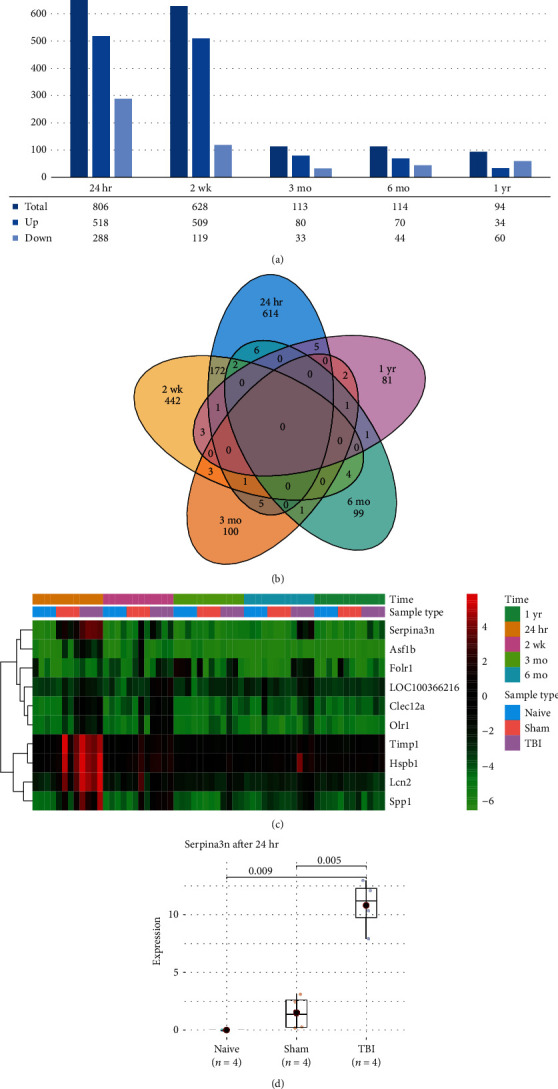
The differentially expressed genes (DEGs) in traumatic brain injury (TBI). (a) The number of DEGs in TBI at the five time points. (b) The Venn diagram for the DEGs shared between the five time points. (c) The expression profiles of the top 10 DEGs with the highest statistical significance. The color band on the top represents the time point and sample type, respectively. (d) The expression levels of Serpina3n in the three groups.

To identify the genes dysregulated by traumatic brain injury (TBI), we collected gene expression data of the rat cortex, which were obtained at 24 hours, 2 weeks, 3 months, 6 months, and 1 year after injury, and their corresponding negative controls (naïve group) with four replicates. Specifically, we identified 806, 628, 113, 114, and 94 differentially expressed genes (DEGs) at the five time points, respectively (Figure 1(a)). The number of DEGs were decreased over time. Moreover, the 24-hour and 2-week time points shared much more DEGs than the other time points (Figure 1(b); Fisher's exact test, *P* value < 0.001), suggesting that the shorter the time interval, the more similar the gene expression profiles. In contrast, the medium to late terms, from 3 months to 1 year, shared much fewer DEGs, suggesting that the gene expression profiles were dynamically changed after traumatic brain injury over time.

Furthermore, we identified the top 10 DEGs ranked by the *P* values, including Serpina3n, Asf1b, Folr1, LOC100366216, Clec12a, Olr1, Timp1, Hspb1, Lcn2, and Spp1 (Figure 1(c)). Notably, Serpina3n achieved the highest statistical significance and was observed to be upregulated in TBI groups at both early and medium terms (Figures 1(c) and 1(d)). These results indicated that Serpina3n might act as a critical regulator in TBI.

### 3.2. Identification of Functional Modules Involved in TBI

**Figure 2:**
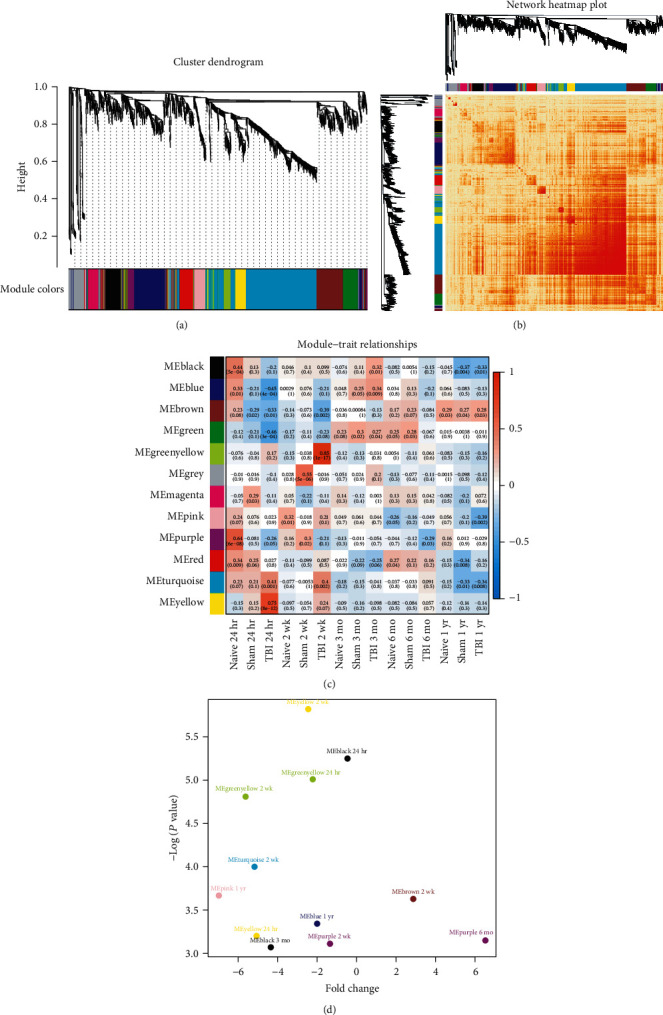
The WGCNA modules identified by the DEGs in TBI. (a) The cluster dendrogram for the DEGs in TBI. The color bars on the bottom represent the modules. (b) The heat map for the similarities of the DEGs and corresponding modules. The red color represents high similarity. (c) The correlation matrix for the module and traits. (d) The volcano plot for the hub genes of the functional modules. The *x*-axis and *y*-axis represent the log2 fold change and -log10 (*P* value).

As the genes could cooperate with each other and be coexpressed as a functional module, we then constructed a weighted gene coexpression network to identify the functional modules using weighted gene coexpression network analysis (WGCNA), which can be used for finding clusters (modules) of highly correlated genes. Specifically, we identified 12 functional modules from the DEGs by WGCNA (Supplementary Table S1, Figure 2(a)). Notably, the genes within each module showed highly similar expression patterns (Figure 2(b)). To further associate the functional modules with the characteristics of the samples, we conducted correlation analysis, and observed that the yellow, turquoise, green, and brown modules were highly correlated with TBI at both 24 hours and 2 weeks, while these modules were rarely correlated with TBI at medium to late terms (Figure 2(c); *P* value < 0.05). The differential expression analysis of the eigen genes revealed that modules including yellow, green yellow, turquoise, black, brown, purple, and pink were highly dysregulated in TBI samples as compared with the controls (Figure 2(d); *P* value < 0.05).

### 3.3. Functional Characterization of the WGCNA Modules

**Figure 3:**
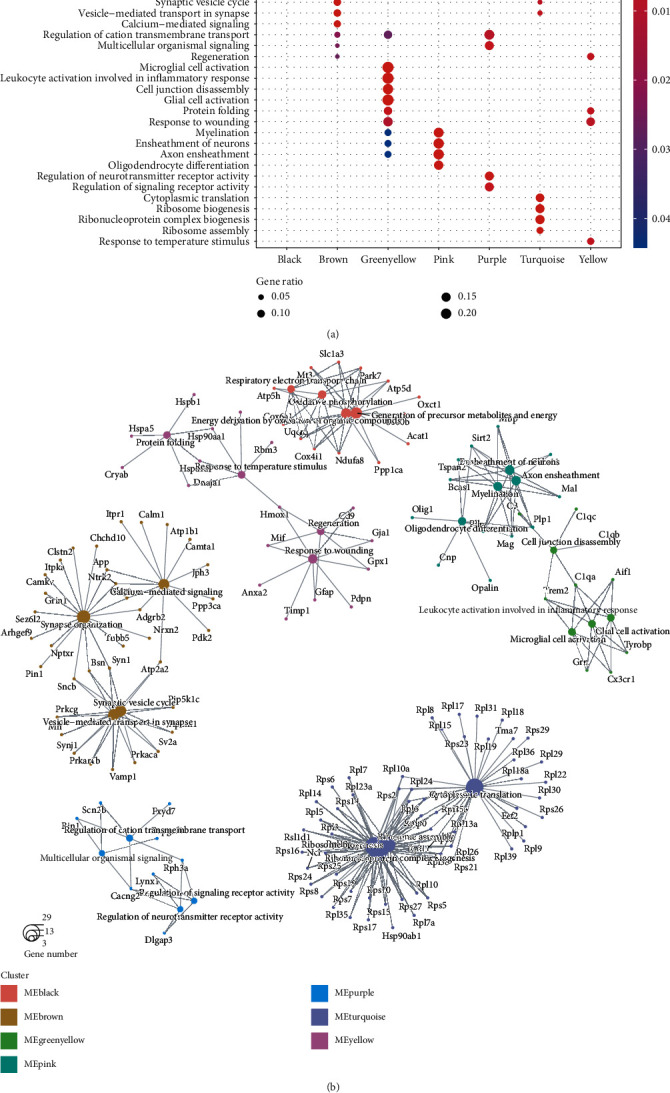
Biological function of the WGCNA modules. (a) The biological processes enriched by the genes within the functional modules. The node color and size represent the adjusted *P* value and the ratio of the number of DEGs to that of the genes involved in the biological process (b).

To further characterize biological function for the WGCNA modules, we conducted gene set enrichment analysis on the genes within the 7 TBI-related modules. Specifically, the black and turquoise modules were mainly involved in energy metabolism and protein translation (Figure 3(a)). The green yellow and yellow modules were related to response to wounding (Figure 3(a)). Moreover, the brown, pink, and purple modules were characterized by nervous system-related biological function (Figure 3(a)). Notably, the genes encoding ribosome proteins like RPL and RPS family genes were frequently identified in the turquoise module (Figure 3(b)). Hmox1, Mif, Anxa2, Timp1, Gfap, Cd9, Gja1, Pdpn, and Gpx1 were involved in the response to wounding, indicating that these genes might be the responses to TBI (Figure 3(b)). The green yellow module highlighted genes involved in microglial cell activation such as Tyrobp, Cx3cr1, Grn, Trem2, C1qa, and Aif1 (Figure 3(b)), suggesting that these genes were responsible for the inflammatory response caused by TBI.

### 3.4. Prediction of Disease Phenotypes by the Hub Genes within the Module

**Figure 4:**
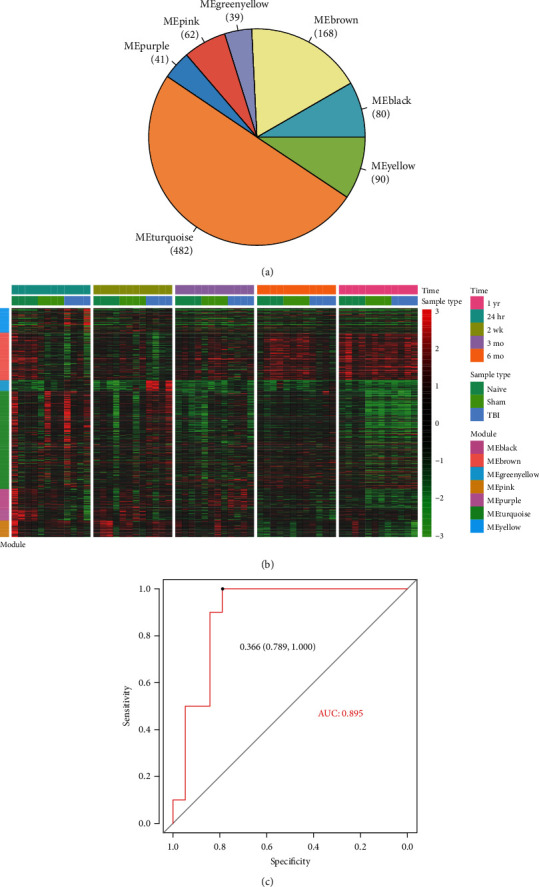
The TBI-related functional modules. (a) The gene numbers for the 7 TBI-related functional modules. (b) The gene expression profiles of the 7 TBI-related functional modules. (c) The receiver operating characteristic (ROC) curve for the random forest classifier.

As shown in Figure 4(a), a total of 962 genes were included in the seven modules. Particularly, yellow and brown modules were upregulated and downregulated at the early term, respectively, but returned to normal at the medium to late term (Figure 4(b)). The turquoise module was upregulated at the early and medium terms, but returned to normal at the late term. In addition, the pink module was observed to be upregulated at 6 months, but downregulated at 1 year. These results indicated that the TBI-related modules were dynamically changed over time.

As the genes within the 7 modules exhibited significantly different expression patterns between TBI and controls, we then tested their separating ability for TBI and control samples. The samples were randomly divided into training (*n* = 30) and testing (*n* = 30) sets, and a classifier was built based on the hub genes in the training set. The classifier achieved a higher area under curve (AUC) at 0.895 about the TBI prediction in the validation set (cut − off = 0.366). These results indicated that the hub genes were closely associated with TBI.

### 3.5. Microglial Cell Activation Is Responsible for Inflammatory Response at the Early and Medium Terms after TBI

**Figure 5:**
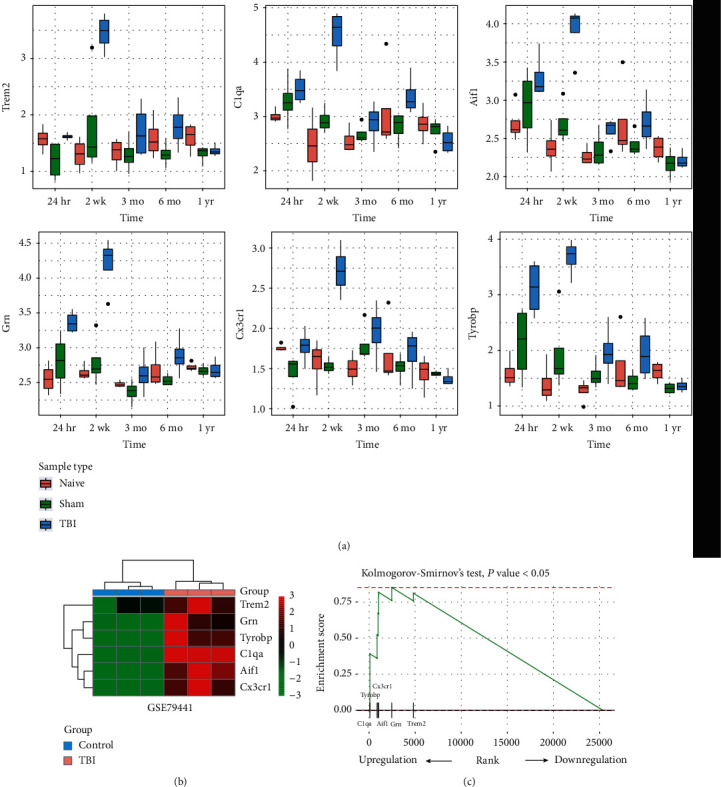
The genes involved in microglial cell activation. (a) The expression patterns of the six genes involved in microglia cell activation following TBI. (b) The validation of the six genes in the GSE79441 dataset. (c) The rank of the six genes by the *t*-statistics evaluating the differential expression level in the GSE79441 dataset.

As microglial cell activation was enriched by the genes within the green yellow module, we then tested the expression patterns of key genes involved in microglial cell activation and inflammatory response. Specifically, we observed that the key genes including Trem2, C1qa, Aif1, Grn, Cx3cr1, and Tyrobp were consistently upregulated at the early and medium terms (Figure 5(a); *P* value < 0.05). To confirm this finding, we collected an independent gene expression data with 3 TBI and 3 control samples. Consistently, the six key genes were upregulated in TBI samples as compared with controls (*P* value < 0.05; Figure 5(b)). Moreover, these genes were highly clustered at the upregulated genes by TBI (Kolmogorov-Smirnov's test, *P* value < 0.05; Figure 5(c)). Furthermore, the six genes were also involved in leukocyte activation and inflammatory response (Figure 3(b)). These results indicated that microglial cell activation and the six key genes were responsible for inflammatory response at the early and medium terms after TBI.

## 4. Discussion

Traumatic brain injury (TBI) is a major cause of morbidity and mortality, both in adult and pediatric populations. However, the dynamic changes of gene expression profiles following TBI have not been fully understood. Specifically, we found that the number of DEGs were decreased over time, and the medium to late terms, from 3 months to 1 year, shared much fewer DEGs, suggesting that the gene expression profiles were dynamically changed after traumatic brain injury over time. Accordingly, the epigenetically dynamic changes have also been observed in brain tissues following TBI [17]. Among the DEGs, Serpina3n, Asf1b, Folr1, LOC100366216, Clec12a, Olr1, Timp1, Hspb1, Lcn2, and Spp1 were identified as the top 10 with the highest statistical significance. Remarkably, Serpina3n has been found as a promising target for neuroinflammation following TBI [18]. Timp1 has been considered as neuroprotective against traumatic and ischemic brain injury in mice [19]. Moreover, Lcn2 (Lipocalin-2) is upregulated by thrombin-induced brain injury through protease-activated receptor-1 activation [20]. These results suggested that these DEGs were highly associated with TBI.

The WGCNA identified 12 functional modules from the DEGs, which showed specific expression patterns over time and were characterized by enrichment analysis. Specifically, the black and turquoise modules were mainly involved in energy metabolism and protein translation. A previous study has shown that glucose metabolism has been altered following TBI [21]. The green yellow and yellow modules including Hmox1, Mif, Anxa2, Timp1, Gfap, Cd9, Gja1, Pdpn, and Gpx1 were related to response to wounding, indicating that expression of these genes such as Hmox1, Anxa2, and Timp1 could protect the brains from brain injury [19, 22, 23]. The green yellow module highlighted genes involved in microglial cell activation such as Tyrobp, Cx3cr1, Grn, Trem2, C1qa, and Aif1, suggesting that these genes were responsible for the inflammatory response caused by TBI. The upregulation of these genes has been validated in a testing dataset. Consistently, Tyrobp and Trem2 were identified as major hubs in human APOE-expressing mice following traumatic brain injury by gene coexpression network [12]. Moreover, Tyrobp and Trem2 may be a promising therapeutic target in TBI. The GRN protein is associated with increased lysosomal biogenesis in activated microglia and can exacerbate neuronal damage after traumatic brain injury [24]. These results indicated that the key genes in microglia cell activation may serve as a promising therapeutic target for TBI.

In addition, the present study also has some limitations such as the small sample size, an independent validation dataset for the dynamic changes of gene expression profiles, and lack of experimental validation. However, the present study still provides a full view of the dynamic gene expression changes following TBI.

## Data Availability

The data used in this study are available at Gene Expression Omnibus (GEO, https://www.ncbi.nlm.nih.gov/gds), which have been cited at relevant places within the text as references.

## Supplementary Materials

Supplementary Materials Supplementary Table S1: the functional modules and their corresponding genes with coexpression.

## References

[1] Wilson L., Stewart W., Dams-O'Connor K. (2017). The chronic and evolving neurological consequences of traumatic brain injury. *The Lancet Neurology*.

[2] Sell S. L., Johnson K., DeWitt D. S., Prough D. S. (2017). Persistent behavioral deficits in rats after parasagittal fluid percussion injury. *Journal of Neurotrauma*.

[3] Maas A. I. R., Menon D. K., Adelson P. D. (2017). Traumatic brain injury: integrated approaches to improve prevention, clinical care, and research. *The Lancet Neurology*.

[4] Faden A. I., Loane D. J. (2015). Chronic neurodegeneration after traumatic brain injury: Alzheimer disease, chronic traumatic encephalopathy, or persistent neuroinflammation?. *Neurotherapeutics*.

[5] Mouzon B. C., Bachmeier C., Ferro A. (2014). Chronic neuropathological and neurobehavioral changes in a repetitive mild traumatic brain injury model. *Annals of Neurology*.

[6] Smith C., Gentleman S. M., Leclercq P. D. (2013). The neuroinflammatory response in humans after traumatic brain injury. *Neuropathology and Applied Neurobiology*.

[7] Adrian H., Marten K., Salla N., Lasse V. (2016). Biomarkers of traumatic brain injury: temporal changes in body fluids. *Eneuro*.

[8] Boone D. R., Weisz H. A., Willey H. E. (2019). Traumatic brain injury induces long-lasting changes in immune and regenerative signaling. *PLoS One*.

[9] Hummel R., Ulbrich S., Appel D. (2020). Administration of all-*trans* retinoic acid after experimental traumatic brain injury is brain protective. *British Journal of Pharmacology*.

[10] Wang Z.-M., Liu C., Wang Y.-Y. (2020). SerpinA3N deficiency deteriorates impairments of learning and memory in mice following hippocampal stab injury. *Cell Death Discovery*.

[11] Saber M., Kokiko-Cochran O., Puntambekar S. S., Lathia J. D., Lamb B. T. (2017). Triggering receptor expressed on myeloid cells 2 deficiency alters acute macrophage distribution and improves recovery after traumatic brain injury. *Journal of Neurotrauma*.

[12] Castranio E. L., Mounier A., Wolfe C. M. (2017). Gene co-expression networks identify Trem2 and Tyrobp as major hubs in human APOE expressing mice following traumatic brain injury. *Neurobiology of Disease*.

[13] Zhong J., Jiang L., Cheng C. (2016). Altered expression of long non-coding RNA and mRNA in mouse cortex after traumatic brain injury. *Brain Research*.

[14] Ritchie M. E., Phipson B., Di Wu Y. H., Law C. W., Shi W., Smyth G. K. (2015). limma powers differential expression analyses for RNA-sequencing and microarray studies. *Nucleic Acids Research*.

[15] Langfelder P., Horvath S. (2008). WGCNA: an R package for weighted correlation network analysis. *BMC Bioinformatics*.

[16] Yu G., Wang L. G., Han Y., He Q. Y. (2012). clusterProfiler: an R package for comparing biological themes among gene clusters. *OMICS*.

[17] Wong V. S., Langley B. (2016). Epigenetic changes following traumatic brain injury and their implications for outcome, recovery and therapy. *Neuroscience Letters*.

[18] Xi Y., Liu M., Xu S. (2019). Inhibition of SERPINA3N-dependent neuroinflammation is essential for melatonin to ameliorate trimethyltin chloride-induced neurotoxicity. *Journal of Pineal Research*.

[19] Tejima E., Guo S., Murata Y. (2009). Neuroprotective effects of overexpressing tissue inhibitor of metalloproteinase TIMP-1. *Journal of Neurotrauma*.

[20] Mao S., Xi G., Keep R. F., Hua Y. (2016). Role of Lipocalin-2 in thrombin-induced brain injury. *Stroke*.

[21] Li H., Li X., Smerin S. E. (2014). Mitochondrial gene expression profiles and metabolic pathways in the amygdala associated with exaggerated fear in an animal model of PTSD. *Frontiers in Neurology*.

[22] Liu N., Jiang Y., Chung J. Y. (2019). Annexin A2 deficiency exacerbates neuroinflammation and long-term neurological deficits after traumatic brain injury in mice. *International Journal of Molecular Sciences*.

[23] Chen-Roetling J., Song W., Schipper H. M., Regan C. S., Regan R. F. (2015). Astrocyte overexpression of heme oxygenase-1 improves outcome after intracerebral hemorrhage. *Stroke*.

[24] Tanaka Y., Matsuwaki T., Yamanouchi K., Nishihara M. (2013). Increased lysosomal biogenesis in activated microglia and exacerbated neuronal damage after traumatic brain injury in progranulin-deficient mice. *Neuroscience*.

