# Supplementary material for: The Gene Coexpression Analysis Identifies Functional Modules Dynamically Changed After Traumatic Brain Injury
Source: Comput Math Methods Med. 2021 Apr 16;2021:5511598. doi: 10.1155/2021/5511598 (PMC8068551; doi:10.1155/2021/5511598)
Supplement: Supplementary Materials — Supplementary Table S1: the functional modules and their corresponding genes with coexpression. [file 5511598.f1.docx]

Short description for Supplementary Table S1:

**Supplementary Table S1. The functional modules and their corresponding genes with co-expression.**
